# Molecular signatures of in situ to invasive progression for basal-like breast cancers: An integrated mouse model and human DCIS study

**DOI:** 10.1038/s41523-022-00450-w

**Published:** 2022-07-18

**Authors:** Aatish Thennavan, Susana Garcia-Recio, Siyao Liu, Xiaping He, Charles M. Perou

**Affiliations:** 1 Oral and Craniofacial Biomedicine Program, School of Dentistry, University of North Carolina at Chapel Hill, Chapel Hill, NC USA; 2 Lineberger Comprehensive Cancer Center, University of North Carolina at Chapel Hill, Chapel Hill, NC 27599 USA; 3 Department of Genetics, University of North Carolina at Chapel Hill, Chapel Hill, NC 27599 USA; 4 Department of Pathology & Laboratory Medicine, University of North Carolina at Chapel Hill, Chapel Hill, NC USA

**Keywords:** Cancer genomics, Transcriptomics

## Abstract

Ductal carcinoma in situ (DCIS) of the breast is a non-obligate precursor of Invasive Ductal Carcinoma (IDC) and thus the identification of features that may predict DCIS progression would be of potential clinical value. Experimental mouse models can be used to address this challenge by studying DCIS-to-IDC biology. Here we utilize single cell RNA sequencing (scRNAseq) on the C3Tag genetically engineered mouse model that forms DCIS-like precursor lesions and for which many lesions progress into end-stage basal-like molecular subtype IDC. We also perform bulk RNAseq analysis on 10 human synchronous DCIS-IDC pairs comprised of estrogen receptor (ER) positive and ER-negative subsets and utilize 2 additional public human DCIS data sets for comparison to our mouse model. By identifying malignant cells using inferred DNA copy number changes from the murine C3Tag scRNAseq data, we show the existence of cancer cells within the C3Tag pre-DCIS, DCIS, and IDC-like tumor specimens. These cancer cells were further classified into proliferative, hypoxic, and inflammatory subpopulations, which change in frequency in DCIS versus IDC. The C3Tag tumor progression model was also associated with increase in Cancer-Associated Fibroblasts and decrease in activated T cells in IDC. Importantly, we translate the C3Tag murine genomic findings into human DCIS where we find common features only with human basal-like DCIS, suggesting there are intrinsic subtype unique DCIS features. This study identifies several tumor and microenvironmental features associated with DCIS progression and may also provide genomic signatures that can identify progression-prone DCIS within the context of human basal-like breast cancers.

## Introduction

Breast cancer (BC) involves the transformation of the normal breast ducts through a variety of histopathologic recognized precursor non-invasive states into fully transformed malignant tumors^1^. Ductal carcinoma in situ (DCIS) is believed to be a precursor of invasive ductal carcinoma not otherwise specified (IDC), the most common BC histologic type^2^. DCIS comprises 20–30% of BC in the US and worldwide^3,4^. Similar molecular profiles, and DNA clonality commonalities, exist between many DCIS and IDC lending support to the precursor status of DCIS to IDC^5^. However, approximately only 20–40% of DCIS progress to IDC if left untreated, and this progression can be in part predicted by the histological grade of the DCIS^6–8^. These studies highlight that there is a subset of DCIS that are true precursors to IDC, and that features like grade can predict a higher propensity of a given DCIS lesion to turn into an IDC. This DCIS subset is likely enriched in cell populations containing genetic and/or genomic aberrations that increase the risk of malignant progression. However, there is a lack of biological understanding and diagnostic methods to robustly identify progression-prone DCIS beyond grade, thus leading to a present state of clinical consensus of DCIS overtreatment^8,9^.

Based on gene expression, IDC can be subdivided into “intrinsic” subtypes with basal-like subtype showing the worst clinical prognosis^10,11^. DCIS can also be similarly subtyped using gene expression like IDC suggesting a molecular continuum as DCIS progresses into cancer^5,12–14^. Specifically, these studies suggest the existence of basal-like DCIS as a distinct entity that is unique from other DCIS. These studies also highlight that basal-like DCIS to basal-like IDC transition is associated with a microenvironment immune cell changes unlike DCIS-IDC transitions of other molecular subtypes^12^. However, PDX models fail to include the complete microenvironment changes in DCIS progression accurately and thus there is a pressing need to use animal models with intact immune systems for studying DCIS progression. In this regard, the C3(1)/SV40 T-antigen GEM model (henceforth called C3Tag) forms early Mammary Intraepithelial Neoplasia (MIN; DCIS equivalent term in veterinary histopathology) that histologically resembles human DCIS and end-state IDC-like tumors of basal-like subtype, and therefore might be a good model to study progression-prone DCIS^15–18^. Our hypothesis is that specific tumor and/or microenvironmental changes occur that governs DCIS to IDC transformation, and that these changes may be identified in a GEM model and also occur in human DCIS as well. Our aim was to identify these molecular changes in C3Tag MIN (henceforth called DCIS) and IDC-like tumors (henceforth called Tumor) utilizing single cell RNA sequencing (scRNAseq) and analyze these findings relative to human DCIS to identify possible commonalities.

## Results

### Epithelial cell populations identified across C3Tag mammary prepuberty, DCIS, and IDC-like tumor states

**Fig. 1:**
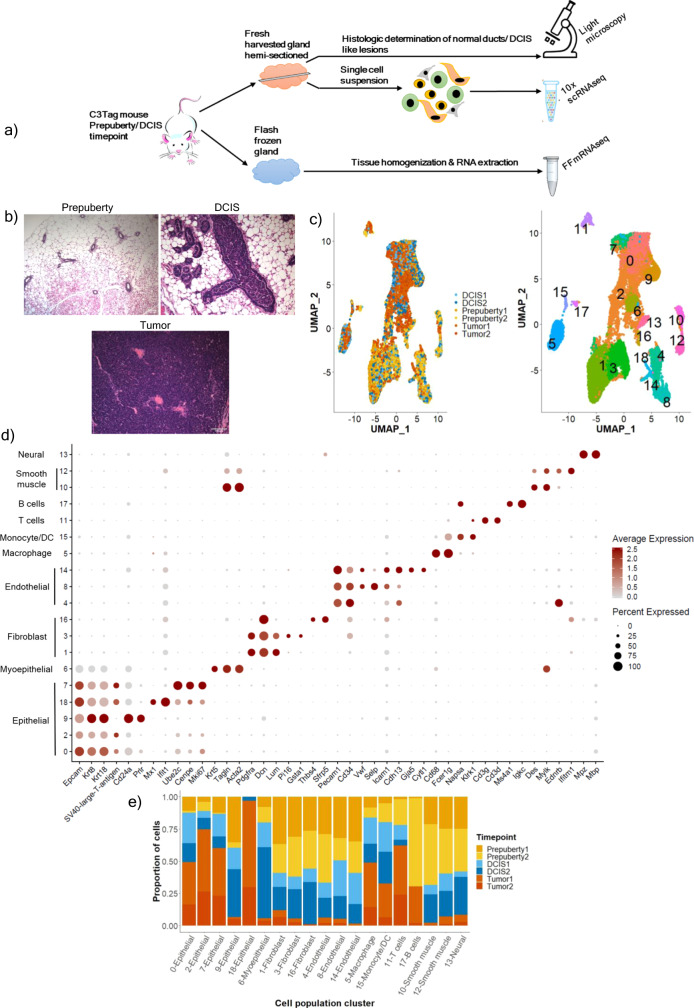
The overall single-cell RNAseq experimental set up and description of all single-cell populations identified per C3Tag disease stage. **a** Schematic of C3Tag experimental strategy to identify MIN/DCIS lesions for downstream scRNAseq and bulk RNA sequencing. **b** Hematoxylin and Eosin (H&E) stained photomicrographs showing normal C3Tag mammary ducts at prepuberty disease state (×100 magnification), DCIS-like MIN lesions at DCIS disease state (×200 magnification) and mammary tumor cells at tumor disease state (×200 magnification). Scale bars, 100 μm. **c** UMAP plot of 21,332 single cells in C3Tag mammary colored by the disease state (Left panel) and by the identified cell populations (Right panel). **d** Dot plot of the expression of specific marker genes across the cell populations identified. **e** Barplots showing relative contribution of disease state to the identified cell populations [MIN: Mammary intra-epithelial neoplasia; DCIS: Ductal carcinoma in-situ, FF-mRNAseq: Flash-frozen mRNA sequenced]. Source data are provided as a Source Data File 1.

To identify both epithelial and microenvironment cellular changes associated with normal ducts transitioning into invasive tumors in the C3Tag mouse model, we performed 6 scRNAseq experiments on the whole mammary glands from three distinct disease states/timepoints (*n* = 2 for each timepoint) including: (1) Prepuberty: 5–6 weeks; (2) DCIS: 12–16 weeks; and (3) Invasive IDC-like Tumor: more than 16 weeks with presence of a palpable tumor. For the gland harvested for DCIS and prepuberty disease state scRNAseq, we hemi-sectioned the mammary gland and performed a routine formalin-fixed paraffin embedded (FFPE) hematoxylin and eosin (H&E) staining to confirm that the gland contained MIN/DCIS lesions and normal ducts before conducting the scRNAseq experiment on the contralateral gland of the same mouse (Fig. 1a). At the same time, we also harvested another mammary gland of the same mouse to perform bulk RNAseq from the prepuberty and DCIS disease states (Fig. 1a). We defined prepuberty and DCIS disease states as per established developmental timepoints for normal ductal and MIN lesions found in C3Tag mouse model^15,19^. Normal ducts are seen in the C3Tag mouse when the *SV40-large T-antigen* is not fully activated and therefore the mice from these time points were collected before they attained puberty and thus called “Prepuberty” state. “DCIS” disease state was defined as the time frame after puberty and where the ducts start containing MIN lesions that are veterinary pathological entities like human DCIS. Furthermore, for this time point we sampled mammary glands that showed higher grade of MIN and presence of areas of central necrosis like human DCIS by routine H&E staining (Fig. 1b). For “Tumor” disease state, palpable tumor was detected, harvested, and sectioned for scRNAseq and bulk RNAseq.

For our primary analyses of C3Tag scRNAseq we captured 21,332 cells from 6 scRNAseq experiments and identified multiple cell type specific clusters comprising the mouse mammary gland milieu (Fig. 1c). We identified statistically significant differentially expressed (DE) genes defining each cluster and used previously determined marker genes to annotate the UMAP cell group clusters. Clusters 0, 2, 9, 18, and 7 were epithelial (*Epcam, Krt8 and Krt18* high; Fig. 1d; Supplementary File 1). We also mapped the *SV40-large-T-antigen* gene sequences on our scRNAseq data and found that clusters 0, 2, 18, and 7 expressed this feature with a significant high expression in cluster 2 (Fig. 1d). Moreover, cluster 7 was composed of cells with *SV40-large-T-antigen* expression and many proliferative genes like *Cenpe* and *Mki67* indicating a mitotically active subset of epithelial cells. Epithelial cluster 9 was devoid of *SV40-large-T-antigen*, showed low expression of proliferative genes, and had high expression of luminal genes namely *Cd24a and Prlr* indicating that this population was likely non-tumor normal epithelial cells (Fig. 1d). We also identified myoepithelial cells as a separate cluster 6 (Fig. 1d) based on the expression of *Krt5, Tagln and Acta2*, and no expression of *SV40-large-T-antigen*. In a similar fashion, we were able to identify several cell types of the microenvironment in all 3 disease states: clusters 1, 3, 16 (Fibroblasts: *Pdgfra, Dcn, Lum*); clusters 4, 8, 14 (Endothelial cells: *Pecam1, Cd34*); cluster 5 (Macrophage: *Cd68, Fcer1g*); cluster 15 (Monocyte/ Dendritic cells: *Napsa, Klrk1*); cluster 11 (T lymphocytes: *Cd3g, Cd3d*); cluster 17 (B lymphocytes: *Ms4a1*), cluster 10,12 (Smooth muscle: *Des*) and cluster 13 (Neural Schwann cells: *Mpz, Mbp*).

Although all the cell clusters in the merged data set were found to contain cells from all three disease states, we found that the relative proportions of cells differed across the disease states. For example, both cluster 9 (normal luminal cells) and cluster 6 (myoepithelial cells) were shown to be almost entirely composed of cells from the Prepuberty and the DCIS disease states (Fig. 1e). To assess the statistical significance of the changes in the cellular composition across all 3 disease states, we utilized a generalized linear regression model that accounted for the batch effects and the 2 technical replicates for each state (Supplementary File 1). The results of this analysis showed that there was statistical significance of differences in cell numbers of epithelial clusters 2, 7, and 11 in the Tumor state versus the DCIS (*p* value < 0.001). In fact, the odds of finding a cell from cluster 2 that was high in *SV40-large-T-antigen*, was 8-fold (odds ratio: 8.05) higher in the Tumor state than the DCIS state. Interestingly, the T lymphocyte cluster 11 (odds ratio: 4.46) and macrophage cluster 5 (odds ratio: 1.35) were also significant for an increased odds ratio to be found in the Tumor state. The same analysis between the Prepuberty and DCIS state showed that the fibroblast and endothelial cells to be significantly more in the prepuberty disease state than the DCIS disease state (Supplementary File 1).

### Genomic and cell biological approaches to identify cancer cell populations and epithelial cells across C3Tag prepuberty, DCIS and IDC-like Tumor states

Since gene expression “dropout” events are known to be associated with scRNAseq data, we utilized inferCNV^20–22^ to identify epithelial cells with DNA copy number changes (i.e., tumor cells) in the C3Tag mouse model, as opposed to only using *SV40-large-T-antigen* expression to identify malignant cells. InferCNV identifies Copy Number Aberrations (CNA) from scRNAseq data as chromosomally located regions of common high, or low, gene expression levels, and is considered a robust means of identifying cancer cells in a mix of normal and cancer cells. InferCNV identifies regions of CNAs using comparisons to normal reference cells, and hence we utilized our previously published scRNAseq data (*n* = 2) of FVB/NJ normal (12 weeks) mouse mammary glands^23^, which are the same strain background as C3Tag mice used here; specifically, we utilized the FVB normal mammary gland epithelial cells to call CNA events in our dataset.

Using the InferCNV approach we were able to identify CNA+ cells in all three disease states (Supplementary Fig. 1), albeit at very different frequencies. Only small numbers of CNA+ cells were identified in our Prepuberty (Supplementary Fig. 1a) and DCIS disease states, with shared events between the two DCIS samples identified (Supplementary Fig. 1a). However, there were distinct human breast CNA events identified in the two Tumor samples where one tumor – ‘Tumor1’ was identified as KRAS altered with a chr6 related KRAS amplification (Supplementary Fig. 1a), which is a previously noted common event in this tumor model^24^. We also performed array CGH (aCGH) analysis on bulk DNA harvested from DCIS and Tumor states and validated many of the CNA profiles coming from the scRNAseq InferCNV calls (Supplementary Fig. 1b, c). For DCIS states, chr2q deletion (del) was identified in both scRNAseq and bulk aCGH. For Tumor, chr1 amplification (amp), chr3 amp, chr6 amp, and chr10 del were identified in bulk aCGH and inferCNV (Supplementary Fig. 1c). Lastly, to robustly identify malignant CNA+ cells for downstream analyses, we used a correlation approach and calculated two correlation values for each cell in relation to i) the CNA profile of the top 5% of non-epithelial cells in each individual disease timepoint and ii) the CNA profile of normal FVB epithelial cells (Supplementary Fig. 2a–e). Cells were designated as CNA high cancer cells if they had a higher score than the median correlation value to the top 5% of non-epithelial cells and had a score lower than the median correlation value to normal epithelial cells. We also looked at the *SV40 large -T-antigen* expression in our CNA inferred cancer cells and found that 80–90% of these cells were *SV40-large-T-antigen* positive. Thus, we identified 2025 CNA high cancer cells (Prepuberty 1: 62 cells; DCIS 1: 319 cells; DCIS 2: 267 cells; Tumor 1: 986 cells; Tumor 2: 391 cells) out of 9679 epithelial cells from our 6 scRNAseq data combined; no CNA high cancer cells were found in Prepuberty 2.

**Fig. 2:**
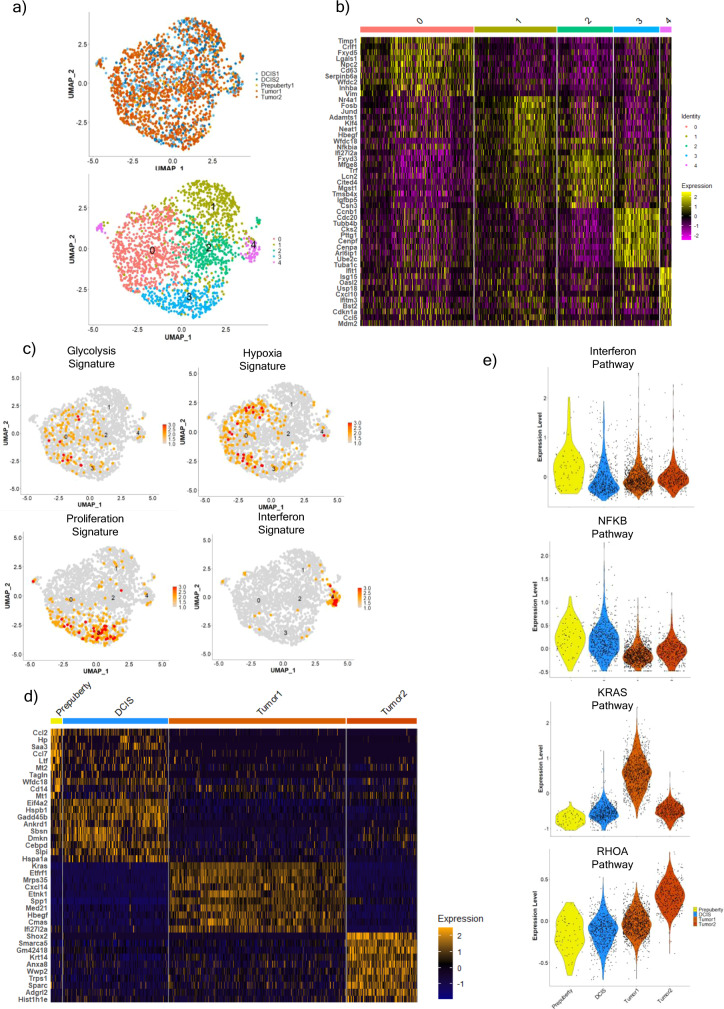
The different subpopulations and gene pathways identified in copy number high C3Tag cancer cells across the disease states. **a** UMAP plot of 2025 copy number high cancer cells with RNAseq data colored by the disease state (Top panel) and by the cell populations identified (Bottom panel). **b** Heatmap of top 10 significant upregulated genes identified per cancer cell subpopulation using the Wilcoxon rank sum test. **c** UMAP plots highlighting significant breast cancer gene signatures from Fan et al.^26^ enriched in specific cancer cell subpopulations: Glycolysis and Hypoxia gene signature for subpopulation 0, Proliferation gene signature for subpopulation 3, and Interferon gene signature for subpopulation 4. **d** Heatmap of top 10 significant upregulated genes identified in cancer cells per Prepuberty, DCIS, Tumor1 (KRAS amplified) and Tumor2 disease states by Wilcoxon rank sum test. **e** Violin plots of NFKB pathway, KRAS pathway, and RHOA pathway scores in cancer cells from Prepuberty, DCIS, Tumor1 (KRAS amplified), and Tumor 2 states. Source data are provided as a Source Data file 2.

**Fig. 3:**
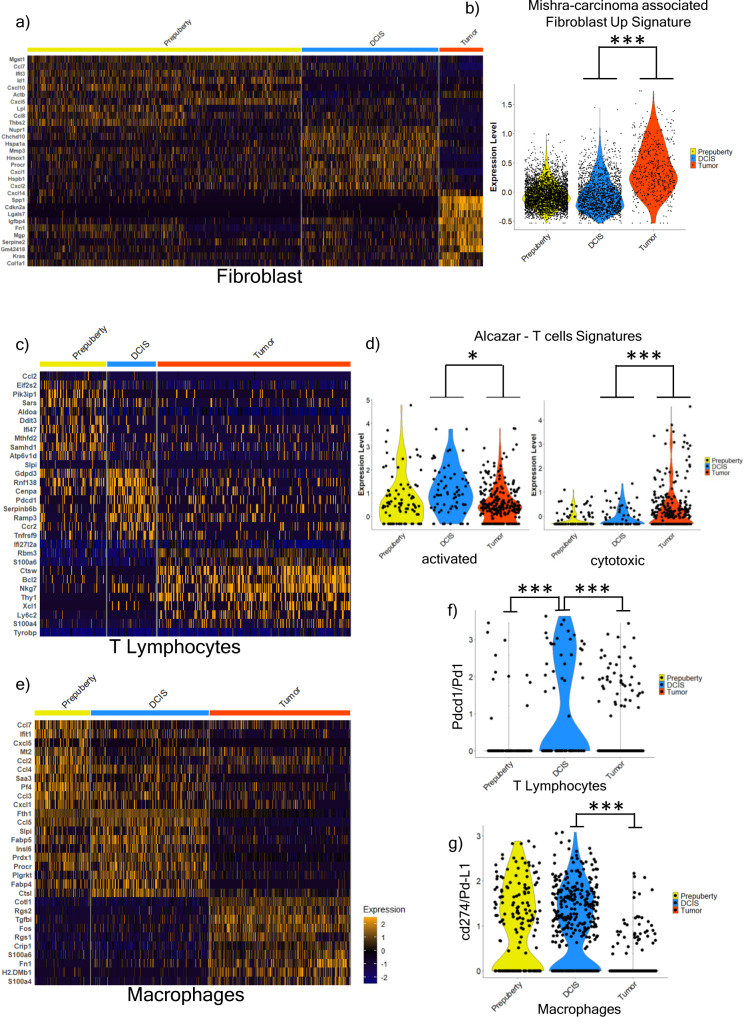
Microenvironment subpopulations and gene signatures across the disease states. **a** Heatmap of top 10 significant upregulated genes identified in fibroblasts per disease states by Wilcoxon rank sum test. **b** Violin plot showing significant enrichment of MSigdbr cancer fibroblast gene signature (Mishra-Carcinoma Associated Fibroblast Up Signature) between C3Tag Tumor fibroblasts (*n* = 564 cells) and DCIS fibroblasts (*n* = 1776 cells) using the t-test with Benjamini–Hochberg (BH) correction. **c** Heatmap of top 10 significant upregulated genes identified in T Lymphocytes per disease states by Wilcoxon rank sum test. **d** Violin plot showing Alcazar et al.^35^ T cell signatures differences between T cells of Prepuberty (*n* = 93 cells), DCIS state (*n* = 69 cells), and Tumor T cells (*n* = 273 cells) by t-test with BH correction. Activated T cell signature (**d**; left panel) and Cytotoxic T cell signature (**d**; right panel). **e** Heatmap of top 10 significant upregulated genes identified in Macrophages per disease states by Wilcoxon rank sum test. **f** Violin plot of Cd274 (human PD-L1) gene in Prepuberty (*n* = 171 cells), DCIS (*n* = 366 cells) and Tumor (*n* = 437 cells) macrophages. *P*-value significance calculated by Wilcoxon rank sum test. **g** Violin plot of Pdcd1 (human PD1) gene in Prepuberty (*n* = 93 cells), DCIS state (*n* = 69 cells) and Tumor T cells (*n* = 273 cells). *P*-value significance calculated by Wilcoxon rank sum test. [*p < 0.05, **p < 0.001, ***p < 0.0001]. Source data are provided as a Source Data file 1.

We next utilized IKAP (Identifying K mAjor cell Pory ducts at prepuberty diseapulation)^25^ to identify the optimal cluster number using the CNA altered cells and identified 5 subpopulations of cancer cells (Figs. 2a, b and 3). Since these are relatively unknown subpopulations, we relied on known breast cancer gene signatures^26^ instead of specific marker genes to identify their biological features. Statistically significant gene signatures indicating unique cellular biological processes were identified: Hypoxia and glycolysis gene signature for cluster 0; Proliferation for cluster 3 and an Interferon/Inflammatory gene signature for cluster 4 (Fig. 2c; Supplementary File 2). Through the gene signature analysis, we were then able to better understand many of the differentially expressed genes identified in these cancer cell subpopulations. For example, cluster 0 had significant high expression of *Aldoa, Pgam1, and Pgk1* (Supplementary File 2), which are involved in the glycolysis pathway, and *Timp1, Ldha, and Eif4ebp1* (Supplementary File 2) that are involved in HIF-1 alpha signaling. Similarly, cluster 4 had significant high expression of *Gbp2, Usp18, Irf7, Ifit1, B2m,* and *Stat1*, which are genes involved in the interferon pathway (Supplementary File 2). Cluster 1 and cluster 2 had no specific enriched breast cancer signatures however cluster 1 had several ribosomal and ER stress-associated genes (*Rpl41, Rps27, Rps29, Fosb,* and *Jund*). Cluster 2 had high expression of other mammary gland-specific genes (*Fxyd3, Trf, Wfdc18, Plekhb1,* and *Lcn2*).

We next identified 141 genes that were constitutively high within all cancer cells in all 3 disease states. These conserved genes comprised predominately of 78 proliferation genes including *Ube2c, Cdc20, and Cenpf* (Supplementary Fig. 4a), and 43 pro-inflammatory genes such as *Cxcl10, Ifit1*, and *Isg15* (Supplementary Fig. 4b; Supplementary File 2). These genes arose early even in InferCNV+ prepuberty cells and remained high in the C3Tag DCIS-Tumor transformation process indicating that these genes could be early markers of cancer transformation. This finding of proliferation-associated genes is also directly related to the natural biology of the *SV40-Large T antigen* that drives tumorigenesis in C3Tag mice by inactivating p53 and Rb. In fact, 30/78 of genes from our proliferation signature had an E2F transcription binding site (Supplementary File 2).

Since we identified many genes aberrantly high in the DCIS state, we sought to examine the panel of OncotypeDx DCIS genes^27,28^ in our mouse models cells dataset. Oncotype Dx DCIS score was specifically developed as a prognostic score to identify biologically aggressive human DCIS and consists of 5 proliferative genes (*Ki67, STK15, Survivin, CCNB1, and MYBL2*), 2 non-proliferation genes (*PR, GSTM1*) and 5 housekeeping reference genes. Interestingly we found 5/7 of the Oncotype Dx DCIS non-housekeeping genes constitutively high in the C3Tag cancer cells from all 3 states except *Pgr*, which was <10% in cells of our murine DCIS states and completely not present in the murine tumor state (Supplementary Fig. 4c); these finding highlights that our CNA high C3Tag cancer cells are expressing genes already used to identify biologically aggressive human DCIS.

We also constructed disease state specific gene signatures for the 3 states. Since both our C3Tag tumors showed different CNA profiles (Supplementary Fig. 1a), they each showed unique upregulated genes with Tumor1 exhibiting high level of *Kras* (Fig. 2d). Cancer cells (i.e., CNA+) from the prepuberty disease state had significant high expression of genes involved in the innate immunity and chemokine signaling pathway *(Ccl2, Ltf, Ccl7, Ccl20*; Fig. 2d). The genes enriched in the DCIS state were associated with regulation of stress response (*Hspa1a, Hspa1b*), apoptosis (*Txnip, Bex3, Gadd45a, and Ankrd1*), proliferation (*Cebpd, Nfkbia*) and inflammation (*Ccl20, F3, Icam1*); however, most genes identified between the cancer cells in prepuberty, and DCIS states were shared in both states (Fig. 2d; Supplementary File 2). We also calculated breast cancer gene signatures for each CNA+ cellular disease state and found many disease state relevant signatures including high expression of interferon signature enriched in prepuberty state; NFKB associated gene signature was enriched in DCIS state; KRAS gene signature was enriched in Tumor1 and RHOA gene signature was enriched in Tumor2 (Fig. 2e; Supplementary File 2). We also computed gene set variation analysis (GSVA)^29^ scores for the MSigDB H: Hallmark gene sets for the cancer cells per each disease states and found similar gene signature patterns (Supplementary File 2). Namely, Prepuberty cancer cells were enriched in Hallmark Interferon alpha response and Hallmark Interferon gamma response; DCIS cancer cells were enriched in Hallmark IL6-JAK-STAT3 signaling and TNFA signaling via NFKB; Tumor1 cancer cells were enriched in Hallmark angiogenesis, KRAS-up and DNA repair; Tumor2 cancer cells were enriched in Hallmark E2F targets and MTORC1 signaling (Supplementary File 2). Thus, using our CNA high cancer cells, we re-identified proliferation gene signatures but also put forth gene signatures that may be associated with basal-like precursor states.

### Microenvironment-specific disease state signatures show similarities between prepuberty/DCIS state and the tumor state

The surrounding fibroblasts and immune cells are considered to play a role in influencing DCIS-Tumor transition^12,30^. We first examined fibroblasts from the 3 disease states and when examined alone these cells clustered into 2 broad populations including a myofibroblast like group (Supplementary Fig. 5a, b; cluster 1) and an inflammatory like group (Supplementary Fig. 5a, b; clusters 0, 2) based upon marker genes from published fibroblasts subsets in BC^31^ (Supplementary File 3). Upon construction of disease state-specific gene signatures, we found that the fibroblasts from the Tumor disease state were distinct (Fig. 3a); these fibroblasts from the Tumor state were associated with increased expression of genes involved in extracellular matrix organization and signaling pathways like Integrin and FGF signaling pathways (*Col3a1, Col5a1, Tnc, Sdc2,* and *Spp1*; Supplementary File 3). We also applied a published breast tumor Cancer-Associated Fibroblast/CAF gene signature^32^ from the Molecular Signatures Database (MSigDB)^33,34^ and found it was significantly higher in the Tumor vs DCIS and Prepuberty fibroblasts (Fig. 3b).

Analysis of immune cells across all 3 disease states revealed 4 broad immune cell types (Fig. 1d and Supplementary Fig. 5c, d), which included T and B lymphocytes, monocytes, and macrophages. Disease state specific T cells signatures showed unique gene expression profiles for DCIS and Tumor states (Fig. 3c; Supplementary File 3). Specifically, DCIS T Lymphocytes exhibited significant upregulation of the *Pdcd1* gene (Fig. 3c, f), which was lower in the Tumor state. We also applied gene signature modules from fractionated T cells from human DCIS and BC^35^ (Supplementary File 3) and noted that the C3Tag mouse DCIS T Lymphocytes had a significant upregulation of activated T cell gene signature (Fig. 3d). Furthermore, we also found a significant upregulation of the cytotoxic T cell gene signature in the Tumor state T cells (Fig. 3d).

Similarly, analysis of macrophages from the 3 states revealed that there were distinct genes in the Tumor macrophages which includes genes like *Cotl1, Tgfbi, Fos,* and *Fn1* along with complement activation genes *C1qa, C1qb,* and *C1qc* (Fig. 3e; Supplementary File 3). Finally, we saw a significant high expression of immune checkpoint markers *PDL1/Cd274* in DCIS macrophages (Fig. 3f) and *PD1/Pdcd1* in DCIS T cells (Fig. 3g), both of which drop in the Tumor state.

### C3Tag DCIS disease state signatures are enriched in Human TNBC and Basal-like DCIS

**Fig. 4:**
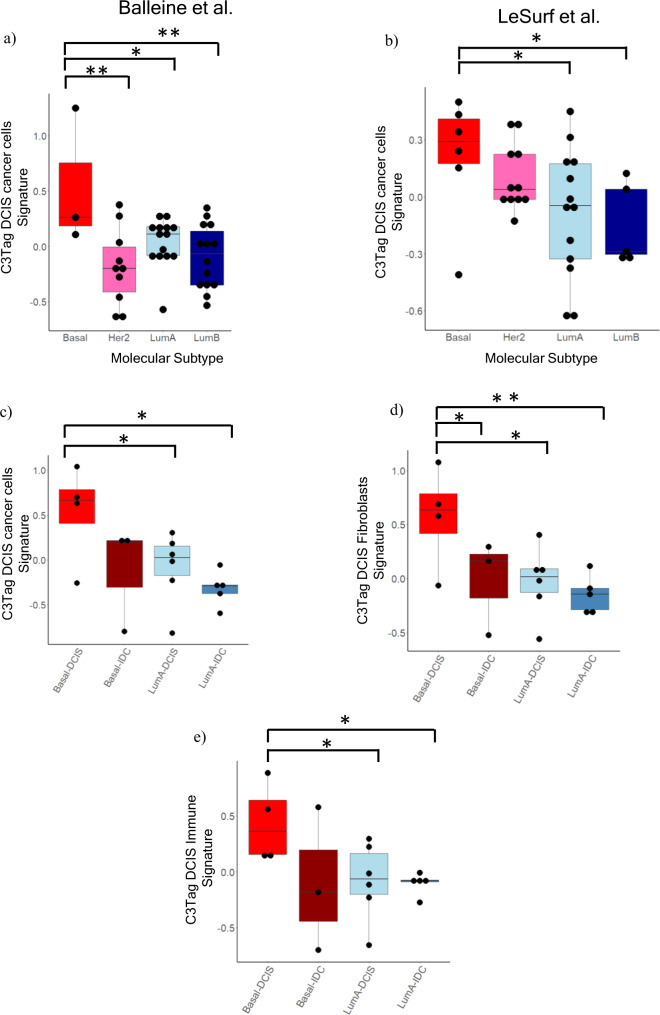
C3Tag gene signatures applied on human basal-like DCIS datasets. **a** Box and whisker plots of DCIS microarray data from Balleine et al.^37^ where *x*-axis denotes the PAM50 subtype and *y*-axis shows C3TAG DCIS cancer cell gene signature. **b** Box and whisker plots of DCIS microarray data from LeSurf et al.^12^ where *x*-axis denotes the PAM50 subtype and *y*-axis shows C3TAG DCIS cancer cell gene signature. **c** Box and whisker plots of Ribo-Zero RNAseq data from human DCIS-IDC tumor pairs where *x*-axis denotes the PAM50 subtype and *y*-axis shows C3TAG DCIS cancer cell gene signature. **d** Box and whisker plots of Ribo-Zero RNAseq data from human DCIS-IDC tumor pairs where *x*-axis denotes the PAM50 subtype and *y*-axis shows C3TAG DCIS Fibroblast gene signature. **e** Box and whisker plots of Ribo-Zero RNAseq data from human DCIS-IDC tumor pairs where *x*-axis denotes the PAM50 subtype and *y*-axis shows C3TAG DCIS immune gene signature. T-test with BH correction was used for all pair-wise comparisons. The upper and lower edges of the boxes represent the upper and lower quartile respectively. The middle line represents the median value. [*p < 0.05, **p < 0.001, ***p < 0.0001]. Source data are provided as a Source Data File 3.

We next sought to evaluate the C3Tag scRNAseq derived gene signatures on human DCIS specimens. To achieve this, we curated multiple published gene expression datasets of human DCIS and performed PAM50 subtyping on all the DCIS samples within these studies. To achieve robustness, we included only published studies that had at least 3 basal-like DCIS samples, which yielded 2 studies^12,36–38^. Since these studies consisted of gene profiles of micro-dissected DCIS epithelial areas, we applied only the C3Tag InferCNV+ malignant cell-derived gene signature onto these human datasets. In both datasets, we found that our C3Tag malignant cells gene signature (Supplementary File 4) was enriched in human basal-like DCIS samples compared to DCIS of other molecular subtypes (Fig. 4a, b).

**Fig. 5:**
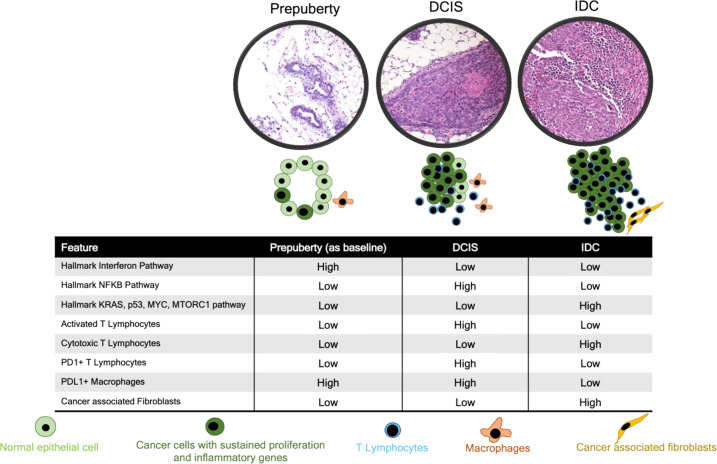
Schematic diagram of DCIS-IDC progression based upon the C3Tag model. The schematic diagram represents key biological pathways and cell population changes from scRNAseq analysis as normal duct (pre-puberty stage) transforms to DCIS, which then transforms to tumor in the C3Tag model. The *interferon pathway* is high in the pre-puberty CNA+ high cancer cells. The *NFKB pathway* also is enriched in the CNA+ high cancer cells in the DCIS state in reference to baseline normal pre-puberty ducts and then becomes low in the tumor state. Tumor specific biological pathways like *KRAS, MYC, p53, MTORC1* are only present in the CNA+ high cancer cells from the tumor and are low in pre-puberty and DCIS states. In terms of the microenvironment cell flux, activated T lymphocytes with increased *PD1*+ expression is highest in the DCIS state. The cytotoxic T lymphocytes are only present in the tumor state. *PDL1*+ macrophages are reduced in the tumor state along with an increased number of cancers associated fibroblasts (CAFs) in the tumor state. All these changes are summarized using pre-puberty normal ducts as the baseline reference. We hypothesize that all these dynamic gene features in multiple cell types along with a sustained proliferation in the CNA+ high cancer cells drive tumorigenesis in the C3Tag basal-like mouse tumor model.

Next, we harvested RNA from archival FFPE DCIS-IDC pairs from our hospital from samples containing synchronous DCIS and IDC within the same specimen; the DCIS and IDC regions for each were individually cored, RNA isolated, Ribo-Zero bulk RNAseq performed, and then we focused on those specimens with PAM50 subtyping characterization of basal-like DCIS (*n* = 4) and basal-like IDC (*n* = 3), LumA DCIS (*n* = 6) and LumA IDC (*n* = 5). Since RNA was collected from tissue sections containing both epithelial and non-epithelial components, we calculated our C3Tag DCIS malignant cells and microenvironment signatures (Supplementary File 4) on these samples. We observed a significant enrichment of the C3Tag DCIS signatures in the basal-like DCIS samples relative to all other DCIS or IDC samples tested (Fig. 4c). We also observed a significant enrichment of the C3Tag DCIS fibroblast signature in human basal-like DCIS (Fig. 4d), and enrichment of C3Tag DCIS immune in human basal-like DCIS (Fig. 4e). All these microenvironment and tumor cell changes are summarized in Fig. 5. Lastly, we investigated NFKB associated gene signatures that were enriched in the CNA+ C3Tag DCIS cells, in all the above human datasets (Supplementary Fig. 6a–c). Basal-like DCIS in 2/3 human datasets (Balleine et al. and the present study) showed a statistically significant upregulation of the C3Tag DCIS-like NFKB gene signature.

## Discussion

It is the current consensus that DCIS is being overtreated leading to physical, emotional, and economic burden for patients and society^39^. This is possibly due to the increased detection of DCIS from increased radiologic screening^40^, and also the knowledge that if left alone, most DCIS would not progress to an invasive disease^6,7^ while yet many DCIS patients receive either systemic and/or local therapies. A challenge in studying DCIS biology is that to study it experimentally one needs to study the dynamic interaction of multiple cells within the controlled environment of a duct through to the occurrence of the invasive disease, or a sufficiently long enough time to know that DCIS will not progress to invasive disease. Currently, many human DCIS basic biology studies utilize MCF10DCIS cell line either alone^41–44^ or injected into a mouse duct (MIND model)^45–48^ to study DCIS in the laboratory. Although these studies add to our biological knowledge, they do not fully mimic human DCIS disease biology as it might interact with the adaptive immune system, which is likely an important component of progression potential. Importantly, a recent study by Risom et al. showed that it is those DCIS with an intact basement membrane and myoepithelial cell activation, and not those with direct tumor-to-microenvironment interactions, that were the most likely to progress^49^; thus, model systems that contain all microenvironment components would be valuable to study DCIS progress. In addition, most human studies utilize human synchronous DCIS-IDC FFPE/frozen tissue to estimate molecular similarities between the two entities, however, these studies link the DCIS features when an invasive component is already present and does not follow the natural course of DCIS progression. Indeed, currently, there are 3 ongoing clinical trials that are studying the progression of low-risk DCIS naturally till the incidence of breast cancer^50–52^. With all these challenges in mind, we used the C3Tag mouse model that spontaneously forms high-grade DCIS-like lesions towards its natural course of forming IDC basal-like mammary tumors^15^.

Here we utilized this consistent murine model, and single-cell RNA sequencing, to study cell type-specific features in the DCIS and invasive disease states, including both tumor cells and non-epithelial cells. Copy number aberrant malignant cells were identified and showed increased expression of genes associated with unique biological pathways including Interferon response in prepuberty state, NFKB pathway in DCIS state, and cancer specific pathways like KRAS, p53, Myc, MTORC1 in the IDC state (Fig. 5). Importantly we also identified in the CNA+ high cancer cells, regardless of disease state, a set of genes with sustained high expression of proliferative and pro-inflammatory genes. For the microenvironment, there was an increase in the number of T cells and macrophages as a normal duct transition to DCIS to IDC, however, there were significant cellular changes in each disease state. There was an increase in PD1+ T cells at the DCIS state in comparison to prepuberty and IDC states. This was also associated with an increased activated T cell signature in the DCIS state (Fig. 5). Conversely there was a reduction of PD1+ T cells and PDL1+ macrophages in the IDC state. The IDC T cells were also more cytotoxic in nature, and it should be noted that the IDC tumors are rapidly increasing in size, and thus the cytotoxic T cells are not keeping the tumor in check despite their presence. Finally, cancer-associated fibroblasts (CAFs) were only found in the IDC state.

Using a methodology of inferring copy number to identify malignant cells from scRNAseq data, we show that gene signatures of glycolysis and hypoxia, along with sustained expression of genes associated with proliferation and interferon pathway, are present in both DCIS and CNA+ tumor/IDC cells. This finding suggests that certain genes and pathways are already initiated in pre-cursor states. Casasent et al. reported the same finding using single cell sequencing on human DCIS-IDC pairs putting forth a polyclonal mechanism of DCIS-invasive transformation^53^. In line with this, we report genes associated with other broad human DCIS pathways like hypoxia^42,54^, glycolysis^54,55^ and proliferation^27^, which have been previously used for mathematic modeling of DCIS progression^54^. Importantly, we recapitulated some of the findings of the prognostic Oncotype Dx DCIS assay and identified 78 proliferation-associated genes sustained in CNA high cancer cells at the C3Tag DCIS stage, including 5/7 exactly found in the OncotypeDX DCIS proliferation feature.

Using our C3Tag DCIS cancer cell data, we report that there is a NFKB pathway enriched in these cells. NFKB has been reported involved in hypoxia and proliferation in breast pre-cursor disease^42,56,57^. Muggerud et al. also reported that the NFKB gene signature was specifically enriched in the ER- high-grade DCIS in comparison to ER+ high-grade DCIS^58^. Liu et al., and Elsarraj et al., have also reported biological mechanisms of NFKB in the DCIS state that can alter the invasive disease course^59,60^. Since we also correlated our C3Tag DCIS cancer cell gene signature to human basal-like DCIS and found that the NFKB gene signatures were significantly high in basal-like DCIS in 2/3 of our human sets, the NFKB pathway activation in the DCIS disease state may play an important role disease progression; additional complex experiments would be needed to definitively demonstrate this hypothesis, although as discussed above, there already exists data in human DCIS implicating the NKFB pathway as being important^42,56,57,59^. One can speculate some technical and/or biological factors that might explain the absence of NFKB gene activation in the basal-like DCIS subset in the Lesurf et al dataset, however, it is difficult to say which it is. These results support additional studies to elucidate the NFKB pathway’s role in DCIS progression.

Applying our C3Tag DCIS cancer cell gene signature on human DCIS datasets, we found that our C3Tag signature was significantly higher in basal-like DCIS versus non-basal-like DCIS, especially the ER+ LumA-like DCIS. Since very few public datasets have gene expression profiling of microenvironment cells from DCIS, we also constructed our own RNAseq dataset from FFPE scrolls of DCIS-IDC synchronous pairs containing cancer and microenvironment cells. Again, upon applying our C3Tag DCIS cancer cell, fibroblast, and immune cell signatures we demonstrate that these are significantly enriched in human basal-like DCIS vs LumA DCIS, thus showing that both tumor cell and microenvironmental features are conserved in human basal-like DCIS and C3Tag mouse basal-like DCIS, and that these are different than what is occurring in Luminal A DCIS.

Although, few studies have examined the microenvironment in human DCIS, both LeSurf et al. and Alcazar et al. reported higher T cells and T cell-based immune signatures in the basal-like DCIS state compared to other molecular subtypes^12,35^. Our C3Tag immune cell findings are similar to their findings including higher number of PD1 expressing T cells in the C3Tag DCIS state. IHC-based studies on pure human DCIS FFPE samples have found that almost all subsets of T cells are increased in ER-negative DCIS^61,62^, but then many go lower in the IDC state. We report a similar decrease in pdcd1(PD1)+ T cells in DCIS vs IDC, however, we also saw an increase in cytotoxic T cell signature in the tumor state and subsequent decrease in PDL1 expressing macrophages in the tumor state, which are features of basal-like invasive breast cancer^61,63^. Recently a study on pure human DCIS has also reported the importance of studying immune-epithelial cell interactions in DCIS and DCIS can exhibit 3 states based on this – active, suppressed, and excluded^64^. This study further highlighted that the exclusion of T cell infiltration in the DCIS duct seen in the excluded state could be seen as an early immune-suppressive event influencing the cancer cells in DCIS to become more aggressive and start showing lack of MHC class I expression^64^. We thereby add to the current knowledge and put forth a specific immune gene signature for basal-like DCIS from immune cells identified in C3Tag DCIS that is loss of PD1+ immune cells as a marker of possible progression, and support more studies in this regard to study immune-epithelial relationships with more spatial methods.

Few studies have analyzed the DCIS-Invasive transition in a subtype-specific manner, but most of them used microarray data from FFPE tissues^12,65^. Our findings strengthen the previous results and put forth new genes of interest. However, we admit that there are limitations to our study. First our mouse tumor single cell analysis may not include all cell types that can be found in the human DCIS setting. Indeed, there might be unique cell subpopulations with other defining gene features that may be present in the human DCIS setting yet not present in our current mouse model. Second, our two C3Tag invasive tumors were molecularly distinct, with each showing unique inferred DNA copy number changes and gene expression features; nonetheless, we were able to find common features between these tumors. Lastly, we only examined a single mouse model, and our sample size of two specimens per time point is noted; however, this does represent >2000 cells per time point per specimen, and thus each specimen was well represented, and each time point showed common tumor cell and microenvironmental features, many of which were also seen in human basal-like DCIS.

In conclusion, we build upon the need to study DCIS based upon a molecular stratification and propose C3Tag mouse model as a good model to study human basal-like progression. We put forth scRNAseq-derived cell type-specific DCIS gene signatures that can be relevant in understanding DCIS biology and clinical behavior, especially since one of our signatures reiterates a major feature of the Oncotype Dx DCIS assay. Finally, we encourage the application of single cell technologies in studying the roles played by cancer cells and microenvironment cells in the malignant transformation of DCIS of other tumor subtypes.

## Methods

### Animal model details

All animal work was carried out in University of North Carolina Division of Laboratory and Animal Medicine (UNC DLAM) facilities in compliance with Institutional Animal Care and Use Committee (IACUC) approved protocols. Female FVB/NJ and C3(1)-Tag mice were obtained in collaboration with the UNC Lineberger Comprehensive Cancer Center (LCCC) Mouse Phase I Unit (MP1U). C3(1)-Tag mice transgenic model produces spontaneous mammary tumors and were originally developed in the FVB/NJ background^15^. Animals were cared for according to the recommendations of the Panel on Euthanasia of the American Veterinary Medical Association. Mice were housed in a climate-controlled Department of Laboratory Animal Medicine facility with a 12 h light:dark cycle and ad libitium access to food and water^66^. The C3(1)-Tag mice are maintained on 2018 Teklad global 18% protein rodent diets (#2918, Harlan/Teklad/Envigo) until tumor development. For C3(1)-Tag mice, the glands were harvested at 5–6 weeks for prepuberty and 12–14 weeks for DCIS stage. The tumor was harvested when it was approximately 1 cm. Animal histopathology was performed by The Animal Histopathology & Laboratory Medicine Core at UNC. Finally, Glands were cryopreserved in liquid nitrogen for bulk RNA isolation.

### Cell Suspension Preparation details for scRNAseq

The mammary glands for prepuberty and DCIS were placed in 10 ml of a digestion medium containing EpiCult™-B Mouse Medium Kit (#05610, StemCell Technologies), Collagenase/Hyaluronidase (#07912, StemCell Technologies), and 1% penicillin-streptomycin (Gibco). The mammary gland was digested overnight in a thermocycler maintained at 37 °C with continuous rotation. The C3(1)-Tag tumors were digested with the Miltenyi tumor dissociation kit (#130-096-730, Miltenyi Biotech) under a gentle agitation setting. The cell pellets retrieved from these suspensions were treated with a 1:4 solution of hanks balanced salt solution (HBSS) and ammonium chloride to remove the RBCs. After RBC removal, the cell suspensions were trypsinized with 0.05% Trypsin and a mix of Dispase and DNAse. A portion of this cell suspension was stained with trypan blue and counted using the Countess Automated Cell Counter (Invitrogen). Based on the counting, the cells were diluted to the appropriate cell stock concentration for running on the 10× chromium machine. Based on the 10× genomics pre-defined cell stock concentrations, each experiment was run to retrieve ~5000 cells after the single cell experiment.

### Single-cell scRNAseq library construction and alignment

The cell suspensions were loaded on a 10× Genomics Chromium instrument to generate single-cell gel beads in emulsion (GEMs) for targeted retrieval of approximately 5000 cells. Single-cell RNA-Seq libraries were prepared using the following Single Cell 3′ Reagent Kits v2: Chromium™ Single Cell 3′ Library & Gel Bead Kit v2, PN-120237; Single Cell 3′ Chip Kit v2 PN-120236 and i7 Multiplex Kit PN-120262” (10× Genomics) and following the Single Cell 3′ Reagent Kits v2 User Guide (Manual Part # CG00052 Rev A). One tumor (Tumor 2) library was processed using Single Cell 3′ Reagent Kits v3: Chromium™ Single Cell 3′ Library & Gel Bead Kit v3, PN-1000092; Single Cell 3′ Chip B Kit PN-1000074 and i7 Multiplex Kit PN-120262 (10× Genomics) and following the Single Cell 3′ Reagent Kits v3 User Guide (CG000183_ChromiumSingleCell3′_v3_UG_RevB). Libraries were run on an Illumina HiSeq 4000 as 2 × 150 paired-end reads. The Cell Ranger Single Cell Software Suite, version 3 was used to perform sample de-multiplexing, barcode ad UMI processing, and single-cell 3′ gene counting. The SV40-large-T-antigen was added as a vector into the Cell Ranger pipeline; the vector sequence is available in SV40-large-T-antigen vector sequence.txt.

### C3Tag mouse sample bulk mRNA-seq library construction and data analysis

Cryopreserved glands/tumors were homogenized using a tissue homogenizer. RNA was isolated using the RNeasy Mini Kit (#74104, Qiagen) according to manufacturer protocol. mRNA quality was assessed using the Agilent Bioanalyzer and libraries for mRNA-seq were made using total RNA and the Illumina TruSeq mRNA sample preparation kit. Paired end (2 × 50 bp) sequencing was performed on the Illumina HiSeq 2000/2500 sequencer at the UNC High Throughput Sequencing Facility (HTSF). Resulting fastq files were aligned to the mouse mm10 reference genome using the STAR aligner algorithm^67^. The resulting BAM files were sorted and indexed using Samtools and quality control was performed using Picard. Transcript read counts were determined was performed using Salmon^68^. Genes with no reads across any of the samples were removed.

### C3Tag array comparative genomic hybridization (arrayCGH) processing and analysis

To investigate DNA copy number changes on bulk tumors, we used the Mouse 244 k Custom Oligo platform (GPL15359 Agilent UNC Perou Lab 1 × 244 k Custom Tiling CGH Array)^69^. Labeling and hybridization were performed according to the manufacturer’s instructions using the Agilent Genomic DNA Labeling Kit PLUS (Catalog Number 5188–5309). One microgram of DNA from liver or spleen of FVB strain mouse was used as normal reference DNA, which was compared versus 1 μg of DNA from C3Tag DCIS and tumor samples. Microarrays were scanned on an Agilent DNA Microarray scanner (G2565CA) and the data uploaded to the University of North Carolina Microarray Database (www.genome.unc.edu). To determine regions of Copy Number Aberration (CNA), we utilized the R package SWITCHdna^18,70^.

### Single-cell scRNAseq preprocessing and data analysis

The 6 scRNAseq Cell Ranger derived output gene-barcode matrices were analyzed and integrated into one single dataset using Seurat R package v.3.0^71^. Individual datasets first underwent a stringent filtering criterion to construct a matrix with relevant genes and cells. For a gene to be selected for downstream analysis, it had to be present in a minimum of 3 cells in the dataset. Similarly, for a cell to be selected, it had to have a minimum of 200 uniquely mapped genes. In addition, dead cells and cell doublets were regressed out by calculating metrics like mito.percentage (mito genes/nUMI) and unique genes mapped ratios (nGene/nUMI). The mito percentage value to exclude dead cells was 5–10. After these filtering steps, the data were ‘log normalized’ and scaled. Variable features were selected according to the default ‘vst’ setting in the Seurat package with *nfeatures* = *2000*. The datasets were then combined into one using the *Seurat:: FindIntegrationAnchors* and *Seurat:: IntegrateData*. Clusters were then identified using 20 significant PCs and visualized as UMAP plots. DE genes were calculated using Wilcoxon rank sum test with a logFC threshold of 0.25 and the top 100 DE genes were calculated for each individual cell subpopulations. Conserved genes were calculated using *Seurat::FindConservedMarkers*.

InferCNV was run using standard settings in ‘sample’ mode of *cutoff* = 0.1, *window_length* = 101 and *max_centered_threshold* = 3. The inferCNV CNA scores were used to calculate correlations in two ways. First, correlation was calculated between the CNA profile of each cell and the average CNA profile of all copy number altered cells within the sample which is similar to the approach by Neftel et al.^20^. Second, correlation was calculated between the CNA profile of each cell and the average CNA profile of all normal cells within the sample. The final cancer cells were identified by plotting the two correlations values for each cell and identifying cells with high correlation to copy number altered cells and low correlation to normal cells. The limits for both correlation scores were identified using the mean +− 2SD.

IKAP was calculated using the Seurat v3 code - https://github.com/NHLBI-BCB/IKAP/tree/master/Seurat3_code for the tumor cell clusters.

Breast cancer gene signatures^26^ were calculated within the single cell gene space by using the Seurat *scaled.data* in the “RNA” assay tab of the integrated datasets. Individual signature values for each cell were calculated as an average expression of all genes present in the gene signature. Once calculated, the significant gene signatures were identified using the Wilcoxon rank sum test.

GSVA was calculated using the log transformed data in the “RNA” assay slot of the integrated datasets in R. We utilized the Hallmark gene sets (H) and the immunologic gene sets (C7) for “mus musculus” using msigdbr R package. Once the GSVA scores were calculated, they were fit into a linear model, and cluster identity, or disease state labels were used to identify significant gene signatures per clusters or disease states.

The generalized linear regression model for cell proportions was constructed using the emmeans R package.

### Human external microarray gene expression PAM50 centroid calculations and data analysis

Microarray data from DCIS studies were downloaded from Balleine et al.^37^ (GSE7882) and LeSurf et al.^12^ (GSE59246). Individual datasets were gene median centered before application of the conventional PAM50 centroid predictions using the 50 gene PAM50 predictor^72^. The median centered values were used for C3TAG DCIS signature calculations. Significance testing was done using t-test with Benjamini–Hochberg correction of p values.

### Human Bulk Ribo-Zero library construction and data analysis

All human tissue was procured under IRB approval from the University of North Carolina at Chapel Hill with written consent from patients to participate. FFPE sections of tumor specimens with co-occurring DCIS and IDC were identified from the medical records, examined by a pathologist, and the DCIS and IDC regions separately cored using 1 mm coring technology typically used to make Tissue Microarrays. Each core was placed into a separate Eppendorf tube and RNA was isolated using the RNeasy Mini Kit (QIAGEN, Hilden, Germany) according to manufacturer protocol. Next, Ribo-Zero libraries were made using Illumina Ribo-Zero plus rRNA Depletion Kit #20037135 following the manufacturer’s protocol. Paired end (2 × 50bp) sequencing was performed on the Illumina HiSeq 2000/2500 sequencer at the UNC High Throughput Sequencing Facility (HTSF). Resulting fastq files were aligned to the human hg38 reference genome using the STAR aligner and transcript read counts were determined was performed using Salmon^67,68^. Genes with no reads across any of the samples were removed. The data were upper-quartile normalized, log-transformed, and median centered before calculating the C3TAG DCIS signatures. Significance testing was done using t-test with Benjamini–Hochberg correction of *p* values.

### Reporting summary

Further information on research design is available in the Nature Research Reporting Summary linked to this article.

## Supplementary information

Supplementary Figures

Supplementary File 1_DE genes for all scRNAseq data defined cell subpopulations

Supplementary File 1_Cell proportion abundance analysis per generalized linear model (GLM)

Supplementary File 1_All cell subpopulation defining specific genes and cell identity labels

Supplementary File 2_DE genes for 5 subpopulations of high CNA+ cancer cells

Supplementary File 2_DE gene signatures (Fan et al 2011) for 5 subpopulations of high CNA+ cancer cells

Supplementary File 2_DE genes for high CNA+ cancer cells per disease state

Supplementary File 2_Sustained inflammatory genes present in high CNA+ cells across disease state

Supplementary File 2_Sustained proliferative genes present in high CNA+ cells across disease state

Supplementary File 2_E2F genes

Supplementary File 3_DE genes for all fibroblasts per disease state

Supplementary File 3_DE genes for all fibroblasts

Supplementary File 3_DE genes for all macrophages per disease state

Supplementary File 3_DE genes for all T cells per disease state

Human T cell gene signatures from Alcazar et al (2017)

Supplementary File 4_Human orthologue genes for the C3Tag constructed gene signatures

Supplementary File 4_Human NFKB gene signatures

Reporting Summary

Dataset 1

Dataset 2

Dataset 3

## Data Availability

All C3Tag mouse 10× single cell RNAseq data generated from the 10× Genomics Cell Ranger pipeline and C3Tag mouse bulk mRNAseq count data are available in GEO database (GSE182389) and raw FASTQs for are deposited in SRA (SRX11865213). All aCGH DNA data are available in GEO database (GSE182389). All the raw human data FASTQs are deposited in dbGAP (phs002443) and in SRA (SRX11865213). Processed human gene counts matrix is deposited in GEO database (GSE182389). The source data underlying Figs. 1c–e, 3a, c, e–g and Supplementary Fig. 5 are provided as Source Data file 1. The source data underlying Fig. 2 and Supplementary Figs. 1–4, are provided as Source Data File 2. The source data underlying Fig. 4 and Supplementary Fig. 6 are provided as Source Data File 3.

## Code availability

There were no special new codes generated for any analysis in this paper. To determine regions of Copy Number Aberration (CNA), we utilized the R package SWITCHdna (version 1.0). All scRNAseq was done by using Seurat R package (version 3.0). Inferred copy number was determined using InferCNV (version 1.10.0). GSVA was calculated using GSVA R package (version 1.41.4) and msigdbr (version 7.4.1). Cell proportion analysis was done using emmeans R package (version 1.5.2). All bulk RNAseq subtyping was performed by PAM50 R functions (https://genome-publications.bioinf.unc.edu/PAM50/).
