# Supplementary material for: Molecular signatures of in situ to invasive progression for basal-like breast cancers: An integrated mouse model and human DCIS study
Source: NPJ Breast Cancer. 2022 Jul 18;8:83. doi: 10.1038/s41523-022-00450-w (PMC9293914; doi:10.1038/s41523-022-00450-w)
Supplement: Supplementary file 1 — Supplementary Figures [file 41523_2022_450_MOESM1_ESM.pdf]

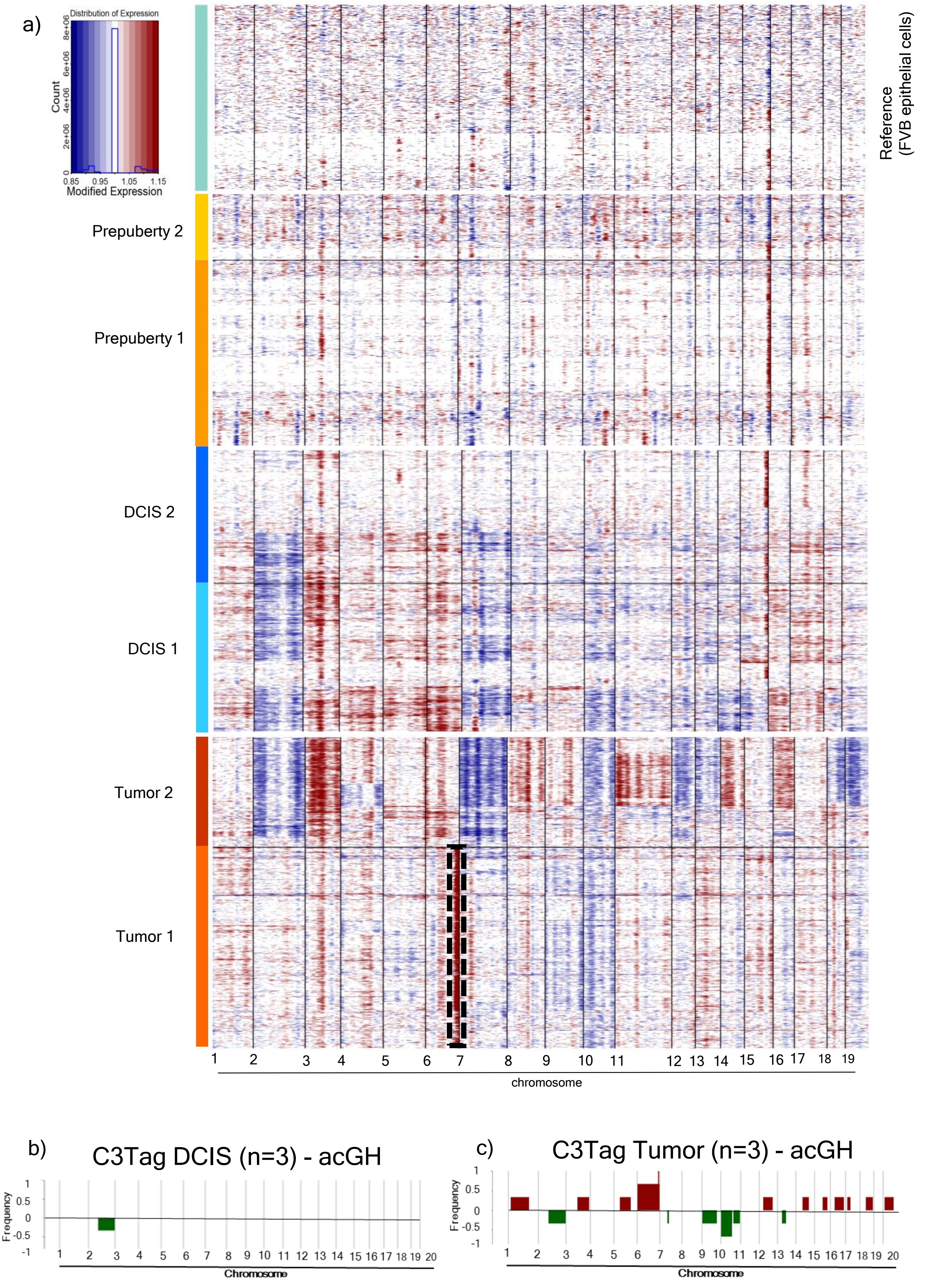

a)
Distribution of Expression
Count
Modified Expression
0.85 0.95 1.05 1.15
Reference (FVB epithelial cells)
Prepuberty 2
Prepuberty 1
DCIS 2
DCIS 1
Tumor 2
Tumor 1
1 2 3 4 5 6 7 8 9 10 11 12 13 14 15 16 17 18 19
chromosome
b)
C3Tag DCIS (n=3) - acGH
Frequency
Chromosome
c)
C3Tag Tumor (n=3) - acGH
Frequency
Chromosome

Supplementary Figure 1. The scRNAseq inferred and bulk copy number events in C3Tag mice (a) InferCNV produced copy number heatmaps of epithelial cells from Prepuberty1 and Prepuberty2**;** DCIS1 and DCIS2 and Tumor1 and Tumor2**.** KRAS amplification is highlighted in the InferCNV plot of Tumor1. **(b)** SWITCH plots identifying copy number segments from aCGH performed on bulk DNA harvested from C3Tag DCIS disease states (n=3). **(c)** SWITCH plots identifying copy number segments from aCGH performed on bulk DNA harvested from C3Tag Tumor disease states (n=3). X-axis represents chromosomes in SWITCH plots and y-axis represents copy number frequency.

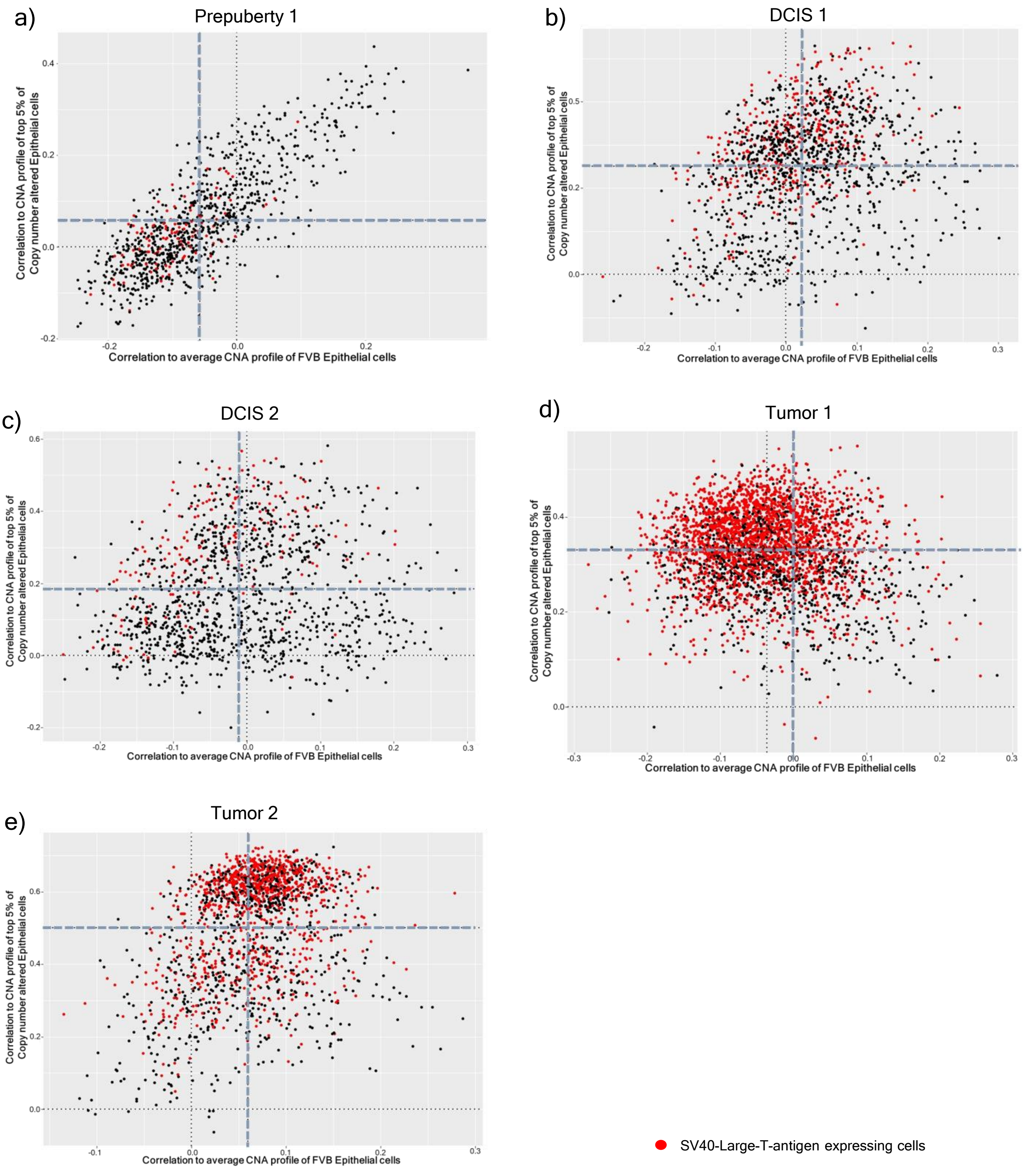

Supplementary Figure 2. The high CNA+ cells identified from scRNAseq inferred CNA profiles (a-e) Correlation plots of InferCNV derived values for Prepuberty1, Prepuberty2, DCIS1, DCIS2, Tumor1 and Tumor2**.** X-axis for individual plot indicates correlation values to the normal (FVB epithelial cells) for all cells of that sample. Y-axis for individual plot indicates correlation values to the top CNA profile of the cells within that sample. Red dots indicate positive expression of the SV40-large-T-antigen. Grey dotted lines indicate the median cutoffs for both correlation scores on x and y-axis indicating the top left quadrant of cells in each individual plot with highest correlation to CNA profile and lowest correlation to the normal cells.

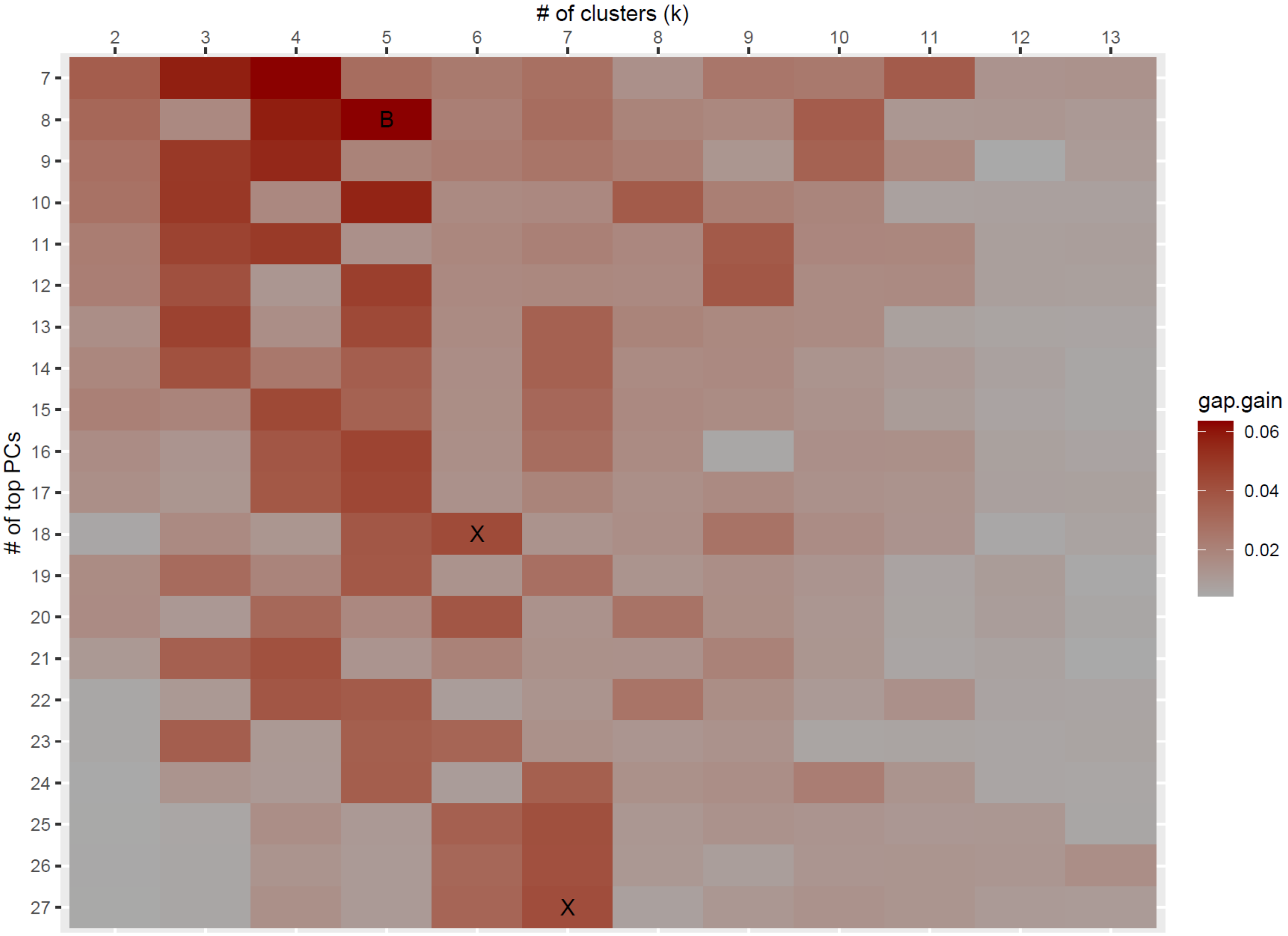

Supplementary Figure 3. Number of possible clusters in high CNA+ cells. IKAP generated plot indicating optimal number of clusters in the InferCNV correlation analysis derived 2025 cancer cell single cells**.** B = Best cluster number; X = Next best cluster numbers.

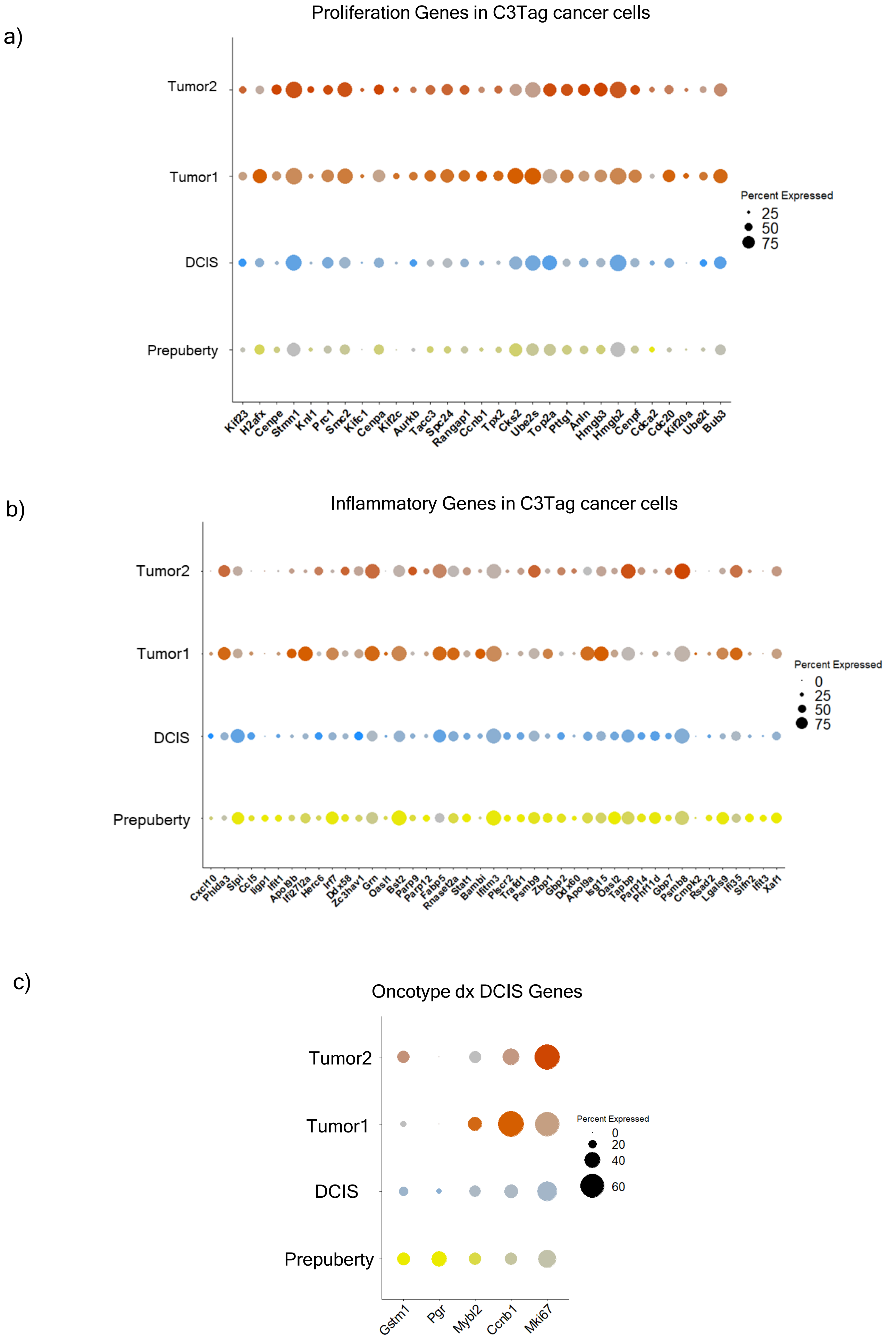
a)
Proliferation Genes in C3Tag cancer cells
Tumor2
Tumor1
DCIS
Prepuberty
Kif23 H2afx Cenpe Stmn1 Knl1 Prc1 Smc2 Kifc1 Cenpa Kif2c Aurkb Tacc3 Spc24 Rangap1 Ccnb1 Tpx2 Cks2 Ube2s Top2a Pttg1 Anln Hmgb3 Hmgb2 Cenpf Cdca2 Cdc20 Kif20a Ube2t Bub3
Percent Expressed
25
50
75
b)
Inflammatory Genes in C3Tag cancer cells
Tumor2
Tumor1
DCIS
Prepuberty
Cxcl10 Phlda3 Slpi Ccl5 Iigp1 Ifit1 Apolgb Ifi27l2a Herc6 Irf7 Ddx58 Zc3hav1 Grn Oasl1 Bst2 Parp9 Parp12 Fabp5 Rnaset2a Stat1 Bambi Ifitm3 Plscr2 Trafd1 Psmb9 Zbp1 Gbp2 Ddx60 Apol9a Isg15 Oasl2 Tapbp Parp14 Phf11d Gbp7 Psmb8 Cmpk2 Rsad2 Lgals9 Ifi35 Slfn2 Ifit3 Xaf1
Percent Expressed
0
25
50
75
c)
Oncotype dx DCIS Genes
Tumor2
Tumor1
DCIS
Prepuberty
Gstm1 Pgr Mybl2 Ccnb1 Mki67
Percent Expressed
0
20
40
60

Supplementary Figure 4. Sustained genes present across the high CNA+ cells across the C3Tag pre-DCIS, DCIS, and Tumor disease states. (a) Dot plot representation of 30 conserved genes associated with proliferation across the disease states**. (b)** Dot plot representation of all conserved genes associated with pro-inflammatory/interferon signaling across the disease states**. (c)** Dot plot representation of 5/7 of the human Oncotype Dx DCIS genes in the 2025 C3Tag cancer cells**.**

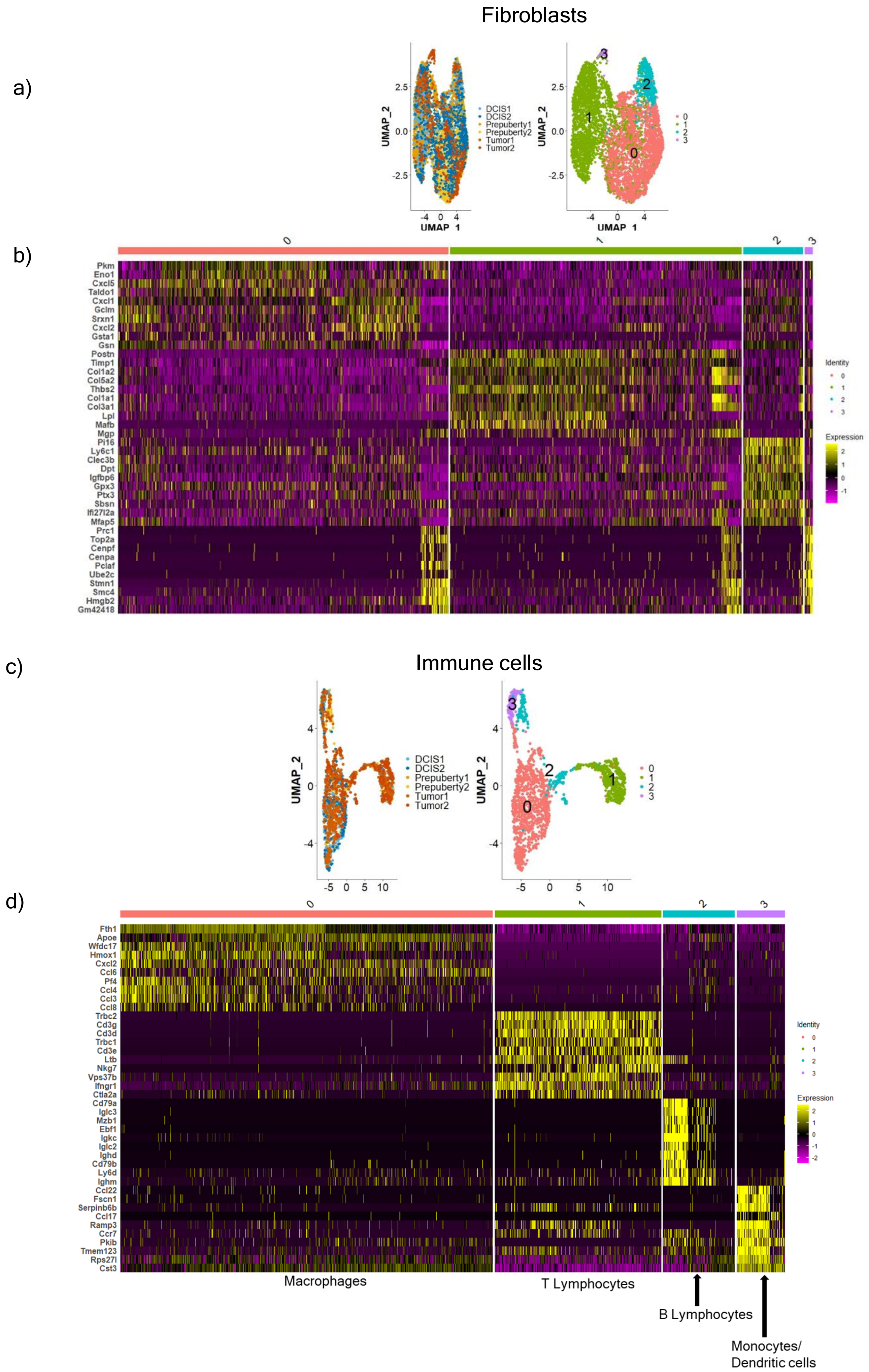
Fibroblasts
a)
UMAP_2
UMAP_1
DCIS1
DCIS2
Prepuberty1
Prepuberty2
Tumor1
Tumor2
0
1
2
3
b)
Pkm
Eno1
Cxcl5
Taldo1
Cxcl1
Gclm
Srxn1
Cxcl2
Gsta1
Gsn
Postn
Timp1
Col1a2
Col5a2
Thbs2
Col1a1
Col3a1
Lpl
Mafb
Mgp
Pi16
Ly6c1
Clec3b
Dpt
Igfbp6
Gpx3
Ptx3
Sbsn
Ifi27l2a
Mfap5
Prc1
Top2a
Cenpf
Cenpa
Pclaf
Ube2c
Stmn1
Smc4
Hmgb2
Gm42418
Identity
Expression
Immune cells
c)
d)
Fth1
Apoe
Wfdc17
Hmox1
Cxcl2
Ccl6
Pf4
Ccl4
Ccl3
Ccl8
Trbc2
Cd3g
Cd3d
Trbc1
Cd3e
Ltb
Nkg7
Vps37b
Ifngr1
Ctla2a
Cd79a
Iglc3
Mzb1
Ebf1
Igkc
Iglc2
Ighd
Cd79b
Ly6d
Ighm
Ccl22
Fscn1
Serpinb6b
Ccl17
Ramp3
Ccr7
Pkib
Tmem123
Rps27l
Cst3
Macrophages
T Lymphocytes
B Lymphocytes
Monocytes/
Dendritic cells

Supplementary Figure 5. scRNAseq profile of individual analysis on C3Tag fibroblasts and immune cells. **(a)** UMAP plot of all fibroblasts with RNAseq data colored according to the disease state **(a; Left panel)** and UMAP plot of all fibroblasts according to cell populations identified by IKAP **(a; Right panel). (b)** Heatmap of top 10 significant upregulated genes identified per fibroblast subpopulation by Wilcoxon rank sum test**. (c)** UMAP plot of all Immune cells with RNAseq data colored according to the disease state **(c; Left panel)** and UMAP plot of all Immune cells according to cell populations identified by IKAP **(c; Right panel). (d)** Heatmap of top 10 significant upregulated genes identified per Immune cells subpopulation by Wilcoxon rank sum test**.** Major Immune populations are annotated based on specific gene markers present in the heatmap.

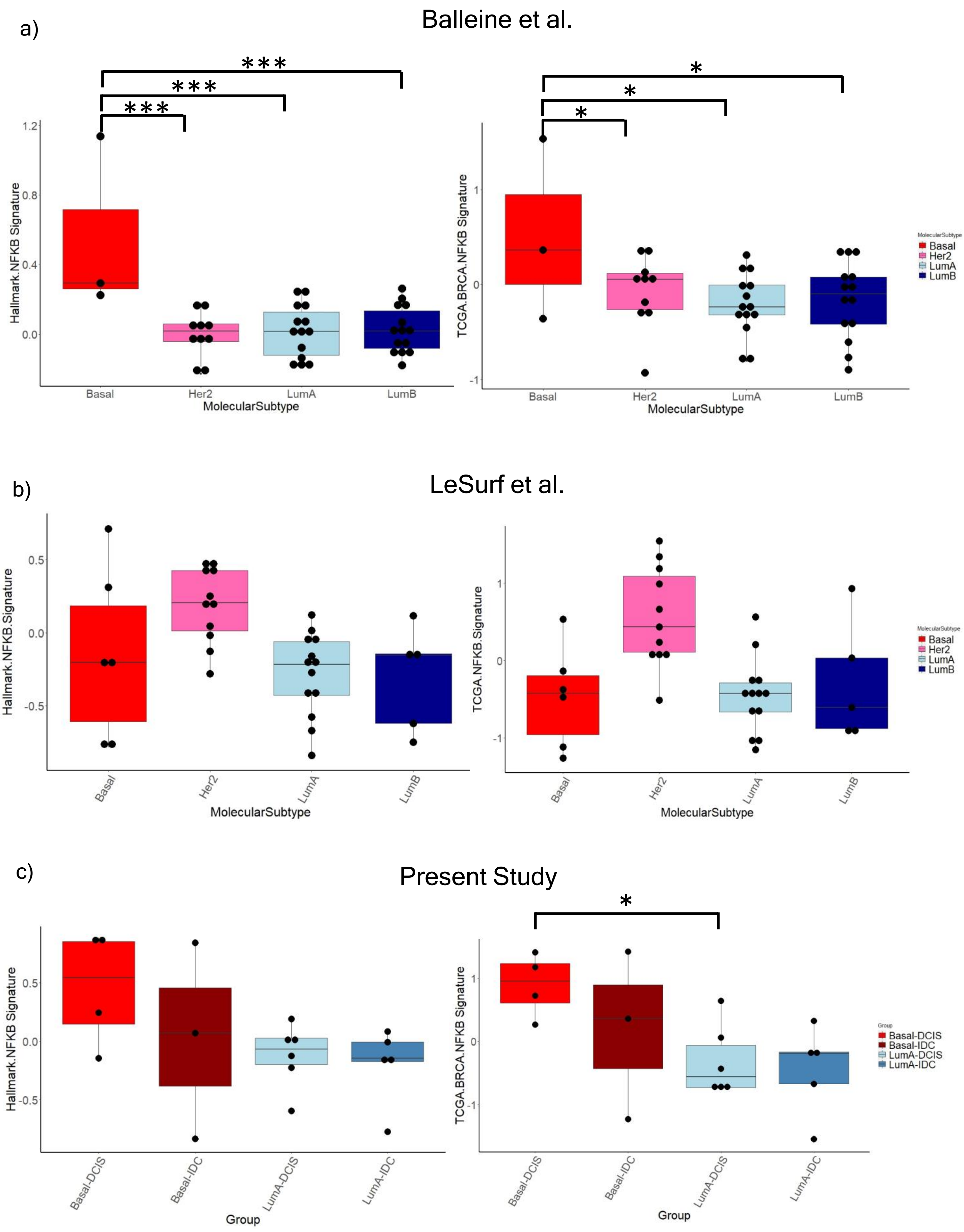
a)
Balleine et al.
***
***
***
*
*
*
Hallmark.NFKB Signature
1.2
0.8
0.4
0.0
Basal
Her2
LumA
LumB
MolecularSubtype
TCGA.BRCA.NFKB Signature
1
0
-1
MolecularSubtype
Basal
Her2
LumA
LumB
b)
LeSurf et al.
Hallmark.NFKB.Signature
0.5
0.0
-0.5
TCGA.NFKB.Signature
MolecularSubtype
c)
Present Study
*
Hallmark.NFKB Signature
TCGA.BRCA.NFKB Signature
Basal-DCIS
Basal-IDC
LumA-DCIS
LumA-IDC
Group

Supplementary Figure 6. NFKB gene signature analysis on human DCIS datasets. (a) Box and whisker plots of DCIS microarray data from Balleine et al[37] where x axis denotes the PAM50 subtype and y axis shows the hallmark NFKB gene signature (left) and TCGA breast cancer NFKB gene signature (right)**. (b)** Box and whisker plots of DCIS microarray data from LeSurf et al[12] where x axis denotes the PAM50 subtype and y axis shows the hallmark NFKB gene signature (left) and TCGA breast cancer NFKB gene signature (right)**. (c)** Box and whisker plots of Ribo-Zero RNAseq data from human FFPE DCIS-IDC tumor pairs of the present study where x axis denotes the PAM50 subtype and y axis shows the hallmark NFKB gene signature (left) and TCGA breast cancer NFKB gene signature (right). T-test with BH correction was used for all pair-wise comparisons. The upper and lower edges of the boxes represent the upper and lower quartile respectively. The middle line represents the median value. [* = $p<0.05$, ** = $p<0.001$, ***=$p<0.0001$]
