# Supplementary material for: Molecular signatures of in situ to invasive progression for basal-like breast cancers: An integrated mouse model and human DCIS study
Source: NPJ Breast Cancer. 2022 Jul 18;8:83. doi: 10.1038/s41523-022-00450-w (PMC9293914; doi:10.1038/s41523-022-00450-w)
Supplement: Supplementary file 10 — Supplementary File 2_E2F genes [file 41523_2022_450_MOESM10_ESM.pdf]

Category	ID	Name	Source	p-value	q-value FDR	Hit Count in Query List	Count in Genc	Hit in Query List
Transcriptior	M29952	E2F5_TARGE	MSigDB	2.30E-13	5.86E-11	30	1273	CDCA3,RANGAP1,CENPA,CENPF,KNSTRN,DLGAP5,PBK,SMC4,HMGB1,MCM2,BIRC5,HMGB2,HMGB3,STMN1,STAT1,CCNA2,PTMS,CDCA2,UBE2T,CCNB2,TPX2,MKI67,GINS2,PLSCR2,BUB3,ALDOA,SKA1,TOP2A,KIF22,AURKB
Transcriptior	M29951	E2F2_TARGE	MSigDB	7.57E-05	2.23E-03	19	1481	CDKN2D,CENPA,INCENP,CENPE,CENPF,HMGB1,HMGB2,SMC2,CCNA2,CCNB1,KIF20A,SPC24,ARL6IP1,TPX2,OASL,CDK1,TOP2A,KIFC1,AURKB
