# Supplementary material for: Molecular signatures of in situ to invasive progression for basal-like breast cancers: An integrated mouse model and human DCIS study
Source: NPJ Breast Cancer. 2022 Jul 18;8:83. doi: 10.1038/s41523-022-00450-w (PMC9293914; doi:10.1038/s41523-022-00450-w)
Supplement: Supplementary file 18 — Reporting Summary [file 41523_2022_450_MOESM18_ESM.pdf]

nature portfolio

Corresponding author(s): Charles M Perou

Last updated by author(s): May 27, 2022

Reporting Summary

Nature Portfolio wishes to improve the reproducibility of the work that we publish. This form provides structure for consistency and transparency in reporting. For further information on Nature Portfolio policies, see our Editorial Policies and the Editorial Policy Checklist.

Statistics

For all statistical analyses, confirm that the following items are present in the figure legend, table legend, main text, or Methods section.

n/a	Confirmed	
☐	☒	The exact sample size (*n*) for each experimental group/condition, given as a discrete number and unit of measurement
☒	☐	A statement on whether measurements were taken from distinct samples or whether the same sample was measured repeatedly
☐	☒	The statistical test(s) used AND whether they are one- or two-sided<br>*Only common tests should be described solely by name; describe more complex techniques in the Methods section.*
☒	☐	A description of all covariates tested
☒	☐	A description of any assumptions or corrections, such as tests of normality and adjustment for multiple comparisons
☒	☐	A full description of the statistical parameters including central tendency (e.g. means) or other basic estimates (e.g. regression coefficient) AND variation (e.g. standard deviation) or associated estimates of uncertainty (e.g. confidence intervals)
☐	☒	For null hypothesis testing, the test statistic (e.g. *F*, *t*, *r*) with confidence intervals, effect sizes, degrees of freedom and *P* value noted<br>*Give P values as exact values whenever suitable.*
☒	☐	For Bayesian analysis, information on the choice of priors and Markov chain Monte Carlo settings
☒	☐	For hierarchical and complex designs, identification of the appropriate level for tests and full reporting of outcomes
☒	☐	Estimates of effect sizes (e.g. Cohen's *d*, Pearson's *r*), indicating how they were calculated

Our web collection on statistics for biologists contains articles on many of the points above.

Software and code

Policy information about availability of computer code

Data collection	No software was used
Data analysis	There were no special new codes generated for any analysis in this paper. To determine regions of Copy Number Aberration (CNA), we utilized the R package SWITCHdna (version 1.0). All scRNAseq was done by using Seurat R package (version 3.0). Inferred copy number was determined using InferCNV (version 1.10.0). GSVA was calculated using GSVA R package (version 1.41.4) and msigdbr (version 7.4.1). Cell proportion analysis was done using emmeans R package (version 1.5.2). All bulk RNAseq subtyping was performed by PAM50 R functions (https://genome-publications.bioinf.unc.edu/PAM50/)

For manuscripts utilizing custom algorithms or software that are central to the research but not yet described in published literature, software must be made available to editors and reviewers. We strongly encourage code deposition in a community repository (e.g. GitHub). See the Nature Portfolio guidelines for submitting code & software for further information.

Data

Policy information about availability of data

All manuscripts must include a data availability statement. This statement should provide the following information, where applicable:

- Accession codes, unique identifiers, or web links for publicly available datasets
- A description of any restrictions on data availability
- For clinical datasets or third party data, please ensure that the statement adheres to our policy

All C3Tag mouse 10x single cell RNAseq data generated from the 10X Genomics Cell Ranger pipeline and C3Tag mouse bulk mRNAseq count data are available in GEO database (GSE182389) and raw FASTQs for are deposited in SRA (SRX11865213). All aCGH DNA data are available in GEO database (GSE182389). All the raw

human data FASTQs are deposited in dbGAP (phs002443) and in SRA (SRX11865213). Processed human gene counts matrix is deposited in GEO database (GSE182389).

Field-specific reporting

Please select the one below that is the best fit for your research. If you are not sure, read the appropriate sections before making your selection.

☒ Life sciences ☐ Behavioural & social sciences ☐ Ecological, evolutionary & environmental sciences

For a reference copy of the document with all sections, see nature.com/documents/nr-reporting-summary-flat.pdf

Life sciences study design

All studies must disclose on these points even when the disclosure is negative.

Sample size	Sample sizes for scRNAseq were chosen based on previous published studies using mouse models and scRNAseq methodology. Sample sizes for human bulkRNAseq was chosen based on a minimum no. of basal-like DCIS samples needed for making statistical comparisons.
Data exclusions	No data is excluded
Replication	All attempts to reproduce the data were undertaken in terms of profiling 2 technical replicates in the scRNAseq experiments and profiling all human basal-like DCIS. To note that the NFKB signature was not found high in basal-like DCIS in one study - Lesurf et al (PMID: 27396337)
Randomization	This is not relevant to the study as all mice conditions were the same and no extrinsic factors were introduced in the study
Blinding	Blinding was not relevant to the study as we are studying the developmental stages on cancer in a mouse model without involvement of any extrinsic factors

Reporting for specific materials, systems and methods

We require information from authors about some types of materials, experimental systems and methods used in many studies. Here, indicate whether each material, system or method listed is relevant to your study. If you are not sure if a list item applies to your research, read the appropriate section before selecting a response.

Materials & experimental systems

n/a	Involved in the study	
☒	☐	Antibodies
☒	☐	Eukaryotic cell lines
☒	☐	Palaeontology and archaeology
☐	☒	Animals and other organisms
☐	☒	Human research participants
☒	☐	Clinical data
☒	☐	Dual use research of concern

Methods

n/a	Involved in the study	
☒	☐	ChIP-seq
☒	☐	Flow cytometry
☒	☐	MRI-based neuroimaging

Animals and other organisms

Policy information about studies involving animals; ARRIVE guidelines recommended for reporting animal research

Laboratory animals	Female FVB/NJ and C3(1)-Tag mice were used. C3(1)-Tag mice transgenic model produces spontaneous mammary tumors and were originally developed in the FVB/NJ background. Three age groups were used: 1) Prepuberty: 5-6 weeks; 2) DCIS: 12-16 weeks; and 3) Invasive IDC-like Tumor: more than 16 weeks with presence of a palpable tumor
Wild animals	This study did not involve wild animals
Field-collected samples	This study did not involve animals from the field
Ethics oversight	All animal work was carried out in University of North Carolina Division of Laboratory and Animal Medicine (UNC DLAM) facilities in compliance with Institutional Animal Care and Use Committee (IACUC) approved protocols. Female FVB/NJ and C3(1)-Tag mice were obtained in collaboration with the UNC Lineberger Comprehensive Cancer Center (LCCC) Mouse Phase I Unit (MP1U)

Note that full information on the approval of the study protocol must also be provided in the manuscript.

Human research participants

Policy information about studies involving human research participants

Population characteristics	Women diagnosed with DCIS at the University of North Carolina at Chapel Hill. FFPE sections of tumor specimens with co-occurring DCIS and IDC were identified from the medical records, examined by a pathologist, and the DCIS and IDC regions separately cored were used. No other clinical information was used or retrieved.
Recruitment	Archival FFPE blocks were used. No additional patients were recruited
Ethics oversight	University of North Carolina at Chapel Hill

Note that full information on the approval of the study protocol must also be provided in the manuscript.
